# Impacts of Health Reform Plan in Iran on Health Payments Distributions and Catastrophic Expenditure

**Published:** 2019-10

**Authors:** Morteza JOSHANI KHEIBARI, Reza ESMAEILI, Mahmood KAZEMIAN

**Affiliations:** 1. Social Determinants of Health Research Center, Gonabad University of Medical Sciences, Gonabad, Iran; 2. Department of Public Health, School of Health, Social Development and Health Promotion Research Center, Gonabad University of Medical Sciences, Gonabad, Iran; 3. Department of Health Economics, School of Medicine, Shahed University, Tehran, Iran

**Keywords:** Health care reform, Health expenditures, Poverty

## Abstract

**Background:**

Health reform in Iran began in 2014, aimed at improving financing pattern of health services. We assessed the reform by changes in variables representing distribution of health payments and catastrophic expenditures.

**Methods:**

Using data from households' income-expenditure survey, this study computed the financial variables, representing poverty line and households at poor state, household's catastrophic health expenditure, fairness in financial contribution (FFC) index, and household's impoverishment state, in the years 2010–2016, in urban and rural areas. The variables were computed by special software designed for this study, based on C-Sharp(C#) programming language, with yearly data on more than 38000 households, each with 1072 information sources.

**Results:**

The food share-based poverty line after sharp rise in 2010–2013, in 2014–2016 raised slowly, and the average percent of households facing catastrophic health expenditure, after sharp rise in 2011–2013, left at 3.25 in 2014–2015 and raised to 3.45 in 2016. The average FFC index remained at 0.839 to 0.837 in 2013–2016. However, interestingly, the average percent of households impoverished after out-of-pocket payments improved from 1.36 to 0.912 in 2013–2016.

**Conclusion:**

In three years of health reform, the major impact of reform was considerable improvements in the rate of the impoverished after out-of-pocket payments. The reform had limited impacts on the rates of households facing catastrophic health expenditure, and on FFC indexes, for the rural and urban residents.

## Introduction

Health system reforms in countries aim at increasing public access to and quality of health care services, as well as equity in health and in utilization of health services (1). Health reforms look for changes in the ways of financing and in the structure of health system in accordance with the aims of qualified and advanced utilization of health services (2, 3). The reforms in the US planned to improve access to affordable health coverage (4). In the UK, the health and social care act in 2012 introduced structural reform that has changed the responsibility of day-to-day running of the NHS (5, 6). In 2009, the reform plan in China with 125 billion dollars budget until 2020, takes five key activities for developing basic insurance, creating national system of necessary drugs, improving primary health care, increasing coverage of primary public health care, and promoting public hospital management (7, 8). Moreover, there is a long list of countries that in the recent decade accepted basic reforms on searching suitable responses for health systems (1, 9).

In Iran, in the recent years, the health system has encountered challenges such as high ratio of out of pocket payment to total health expenditure, and high prevalence rate of non-communicable diseases that required basic reforms in the performance of health system (10). Health reform plan in Iran started in May 2014 with the aim of responding to the existing challenges, especially reduction of direct payments of households at times of obtaining health care services (11). Need to the reform has been emphasized in the 5-year national plans (12, 13). According to the latest plan, a number of government funding resources such as resources of social insurance, subsidy reforms and value-added tax were applied to reduce households' out-of-pocket payments and improve services quality (11, 12).

WHO in its report in 2000 declared fair financial contribution, FFC, was one of the main goals of a health system (14). Equity and FFC in any health system are affected by households' capacity to pay and need-based health care services (15). Evaluation of health system could be shown by the measures of fairness in health financing, Gini coefficient, Kakwani index, FFC Index, catastrophic health expenditure and impoverishment index of health expenditures (16, 17). The WHO emphasized the FFC index as important measure of evaluation of performances of health systems (18, 19). Based on WHO definition, households with catastrophic expenditure spend more than 40% of their capacity to pay or the sum of non-food sources, or above 10% of their total expenditure on health (19,20). These indicators could be used to evaluate the impacts of health system reform plan in Iran. The achievements of this plan could be measured by changes in households' health expenditures after the implementation of the plan, or by changes in FFC indicator, catastrophic expenditure, and impoverishment due to health expenditure, in urban and rural areas. In many countries, these measures have played important role in guiding health reforms, control and supervision (15, 21, 22).

In Iran, the health system reform plan has received considerable government budget from 2014 (11), and this study presented evaluation of it in 3-year period.

## Materials and Methods

This descriptive-analytical examination of the Iranian health reform in the years 2014–2016, regarding the trends of distribution of health payments in 2010–2016, and the methods introduced by WHO (19). The data were collected from the source files of households' income-expenditure surveys in the years 2010–2016, presented by the Iranian Statistical Center, ISC. The whole data sample size in urban and rural areas are shown in Table 1. The questionnaire of the surveys and the method of collecting data in the ISC have been adapted by the Classification of Individual Consumption by Purpose (COIOP) that provide data with international comparisons.

**Table 1:** The sample size of household's cost and income survey 2010–2016

***The sample size of household's cost and income survey***							
Year	2010	2011	2012	2013	2014	2015	2016
Level							
Total	38285	38513	38192	38316	38275	38252	38146
Urban	18701	18727	18535	18880	18885	18871	18809
Rural	19584	19786	19657	19436	19390	19381	19337

The results were computed by the software specially designed for this study that is based on C-Sharp(C#) programming language 1. The software includes all the required survey data of households in the annual samples, accounting for yearly 38252 to 38513 households in the samples in the years 2010–2016, and more than 1072 annual data from the expenditure and socioeconomic questionnaire for each household. This software has the power to provide many expected computations on the distributions of households' income-expenditure data.

To compute the poverty line, pl, and then, the ratio of poor households to total households, we used the food expenditure-ratio approach that avoids complications in the assumptions and in the results from the other approaches to the food share of each household. Following WHO (19), we computed food expenditure share for each household in the sample, and choose the households in the 45th to 55th percentile range. The following equation gives the average food expenditure in the median range, or the pl, using the weights from whole sample data.

(Equation 1) $$\text{pl}=\frac{\sum {\text{W}}_{\text{h}*}{\text{eqfood}}_{\text{h}}}{\sum {\text{W}}_{\text{h}}}$$

where, eqfood_h_ is household's equivalised food expenditure, equal to household's food expenditure, food_h_, divided by the equivalent household size, eqsize_h_ 2, and w_h_ is household weighting variable, introduced by the ISC in households' expenditure survey.

The subsistence expenditure for each household, se_h_, can be obtained from the following equation,

$${\text{se}}_{\text{h}}=\text{pl}*{\text{eqsize}}_{\text{h}}$$

and the result can be used to determine household's state of poor, poor_h_, when household's total expenditure, exp_h_, is less than household's subsistence expenditure, se_h_. That is,

(Equation 2) $$({\text{exp}}_{\text{h}}<{\text{se}}_{\text{h}})\to {\text{poor}}_{\text{h}}$$

Equation (1) and inequality (2) provide estimation of poverty line and the number of poor households, respectively.

WHO definition implies when household's total out-of-pocket health payments, oop_h_, exceed 40 percent of household's capacity to pay, ctp_h_, she faces catastrophic health expenditure. This can be shown by the following inequality.

(Equation 3) {(oophctph)>0.4}→catah

where, cata_h_ represents household at the state of catastrophic health expenditure, and ctp_h_ is taken as household's non-food expenditure or non-subsistence spending, defined by

$$\begin{array}{l} {\text{ctp}}_{\text{h}}={\text{exp}}_{\text{h}}-{\text{se}}_{\text{h}},\;\;\;\;\;\;\;\text{if}\;\;{\text{se}}_{\text{h}}<{\text{food}}_{\text{h}},\\ {\text{ctp}}_{\text{h}}={\text{exp}}_{\text{h}}-{\text{food}}_{\text{h}},\;\;\;\;\;\;\text{if}\;\;{\text{se}}_{\text{h}}>{\text{food}}_{\text{h}}. \end{array}$$

Inequality (3) could be used to compute the number of households facing catastrophic health expenditure.

To compute fair financing contribution, FFC, index as overall inequality in household financial contribution on health, we used the following equation, implied by the WHO.

(Equation 4) $$FFC=1-\sqrt[{3}]{\frac{\sum w_{h}{\left|oopctp_{h}-oopctp^{c}\right|}^{3}}{\sum w_{h}}}$$

where,

oopctph=(oophctph)oopctpc=(∑wh*ooph∑wh*ctph)

The FFC equation (4) weighs heavily the households that spend large share of capacity to pay on health. The FFC ranges between 0 and 1, the faire, the close to 1.

The last financing distribution in this study is concerned with impoverishment. Households may become poor after paying for health care services. This can be shown by impoor_h_ defined by

(Equation 5) $$\left\{\mathrm{ex}p_{h}>se_{h},\;\mathrm{and}\;\mathrm{ex}p_{h}-\mathrm{oo}p_{h}<se_{h}\right\}\to \mathrm{impoo}r_{h}$$

Equation (5) could be used to compute the number of households impoverished after out-of-pocket payments.

## Results

The results obtained from computing the variables of poverty line, *pl*'s, households at the state of poor, *poor_h_*'s, households at the state of catastrophic health expenditure *cata_h_*'s, fair financing contribution, *FFC*, indexes, households impoverishments after paying for health care services *impoor_h_*'s, in the years by definitions are shown in the above equations and inequalities 1 to 5.

**Fig. 1:**
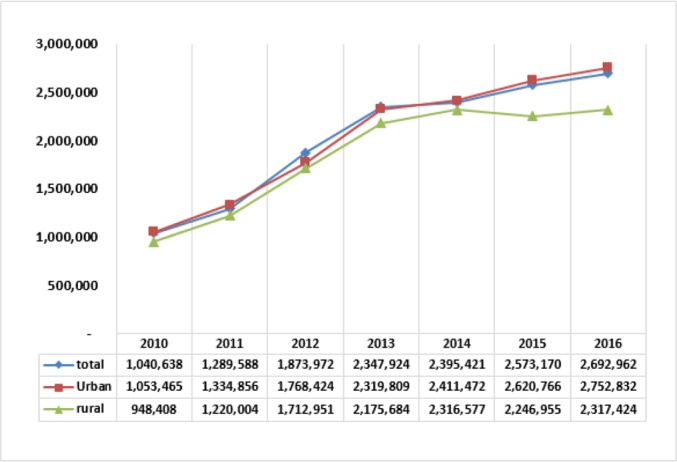
Distribution of food-share based poverty line (Rails)

**Fig. 2:**
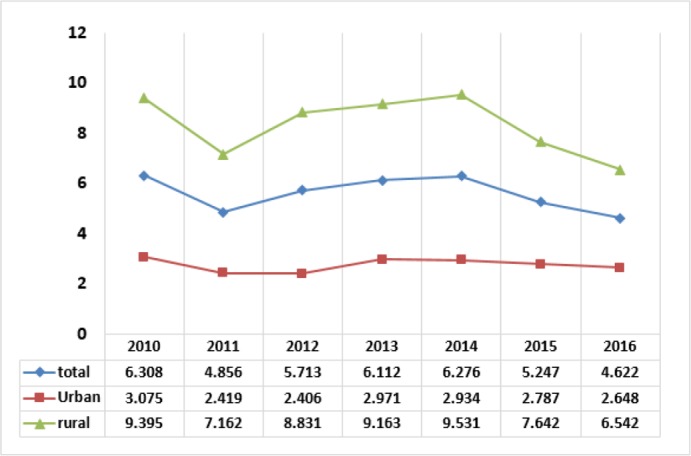
The percent of poor households

Fig. 1 shows the results of changes in the food share-based poverty lines in the years 2010–2016 for urban and rural areas and the country as a whole, totally with rising trends. These show hardness in staying above the poverty line, especially for the people with income at the lowest income margin. This arouse from high inflation rates above 25% in 2011–2013, and close to 10% in 2014–2016, along with small growth rates of average income in these years. Fig. 2 shows the trends of percent of poor people below the poverty line. Since 2011, the average rate of the poor, after reaching the highest level in 2013 and 2014, declines in 2015 and 2016. This is highly important for the rural residents, and less noticeable for the residents in urban areas.

**Fig. 3:**
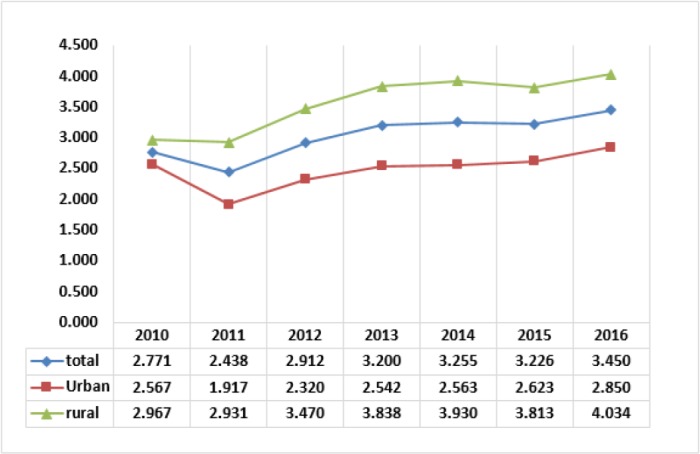
The percent of households facing catastrophic health expenditure

Fig. 3 shows the trends of the percent of households facing catastrophic health expenditure. The trends for the urban and rural residents and on average indicate decline in the intensity of the growing rates of people caught in catastrophic health expenditure in the first and second years of the health reform. However, in the third year, the trends show lack of effects of the reform on the increasing rates of people in the catastrophic state.

**Fig. 4:**
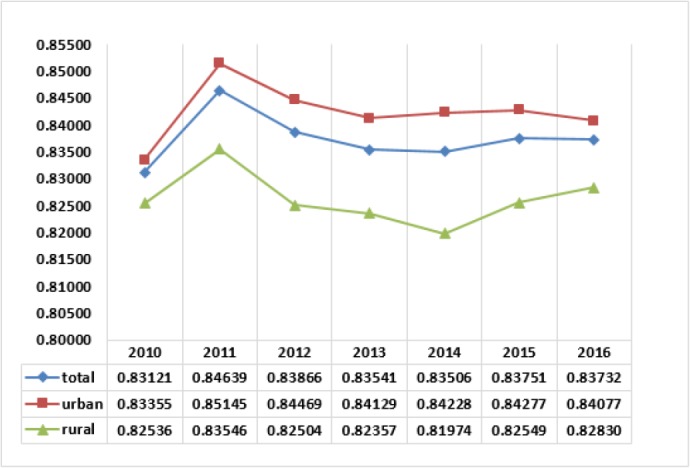
The FFC index representing household financial contribution on health

Fig. 4 shows changes in FFC indexes as overall inequalities in households' contributions in health expenditures, in the years 2010 to 2016. In these years, the best indexes belong to 2011. The trend of average FFC index in the beginning year of reform in 2014, almost stopped declining change, and after a small rise in 2015, almost remained unchanged in 2016. The trend of FFC index for the residents in rural areas improved sharply in the last two years. However, the FFC index for the urban residents after narrow improvements in 2014 and 2015, declined undesirably in 2016.

**Fig. 5:**
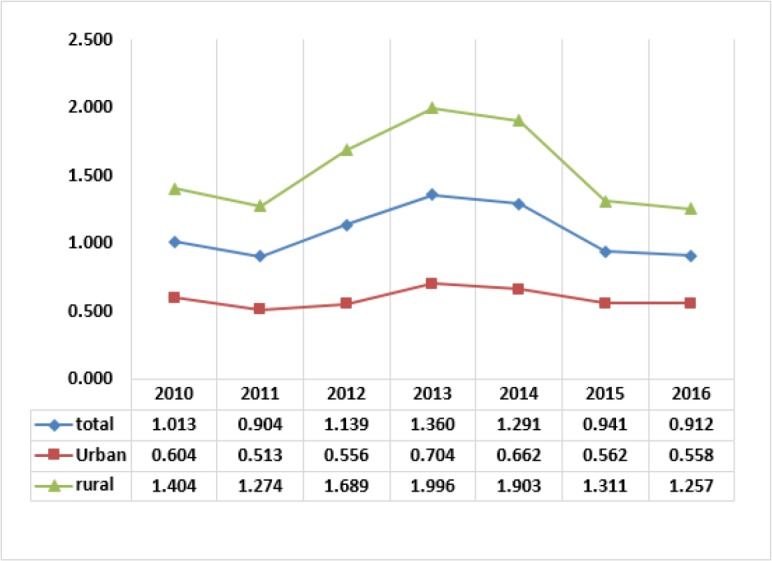
The percent of households impoverished after out-of-pocket payments

Fig. 5 shows the trends of the percent of households impoverished after out-of-pocket payments. After the health reform in 2014, the trends show sharp decrease in the rate of impoverishment, especially in 2015. This is the most exciting result of the reform in the Iranian health system in 2014.

## Discussion

In many countries, there are increasing interests in developing studies on the distribution of health payments and catastrophic expenditures, and guides on upgrading health system payment mechanisms along with reforms in the health system. In study with data from 59 countries, the highest rates of catastrophic health expenditures were found in many developing countries and in Latin America (23). In these countries, catastrophic expenditures could be shown by three key factors of low accessibility of health services, low income of household and lack of universal health insurance coverage. Korean Health Panel Survey in 2008, about 3.5% percent of households were encountered catastrophic health expenditures (24). The impact of implementation of new medical cooperative plans of China was evaluated; this plan was effective on protection of people against catastrophic health expenditures and tuberculosis and impoverishment index was reduced from 16.1% to 7.3% (25). Some other studies investigating fair financing contribution were from Kenya (26), Turkey (27), Colombia (28) and Turkey (21).

In Iran, about 3.38% of households were encountered catastrophic health expenditures and 1.5% were under the poverty line due to the compensation of health expenditures (17). FFC indexes showed higher inequality in rural areas compared to urban areas, and the average FFC index was accounting for 63% for the country as a whole. The FFC index for the Iranian health system was computed in the years 2003–2010, and the FFC index had descending trend during the 8 years (29). The study for the years 1984–2009 showed that the FFC index had low fluctuations in Iran, that is, in rural areas, it changed between 0.76% and 0.75%, and in urban areas, it stood at 0.79% (30). In other studies, some results on FFC were reported in Iran (31–33). All these studies lacked special attention on the effects of health reforms in their studied period.

In this study, it was important to take notices on changes in the rates of the poor, the households facing catastrophic health expenditure, and the households impoverished after out-of-pocket payment, and the FFC index, before and after the health reform in May 2014. In the Iranian health reform, it was aimed to reduce copayments for outpatient and inpatient services in the public hospitals and clinics, and to encourage the public to move in those settings, along with increasing the quality of services in the public health settings. In Iran, more than 80% of the hospitals were managed specially for the health reform plan. This means that the reform started to accomplish great work to improve health payment distributions of the public. This study revealed that improvements happened, but less than that expected.

This study concentrated on changes in financial variables to assess health reform in Iran at country level. The results could be enriched by details at province level, and applying health indicators in the assessment processes. Such enriched assessments could make the financing policies in health reform with the expected consequences on improving the rates of households facing catastrophic health expenditure, and the FFC index, by allocating relatively more resources to the provinces at more needs.

The assessment materials and methods in this study were confined to households' resources and direct payments. Lack of estimates on changes in outpatient and inpatient services at province and country levels, leave sources of expected and/or unexpected results from the reform unanswered.

## Conclusion

In the three years of health reform, the rates of the poor in the urban and rural areas decreased and supported health reform strongly to reduce the rates of households impoverished after outof-pocket payments. However, in these years, slowly rising the rates of households facing catastrophic health expenditure, were found with unpleasantly no considerable effects from the health reform, especially in the last year. The FFC index for the rural residents successfully raised at the two last years of the health reform. However, the average FFC index after a small rise in 2015, remained almost unchanged in 2016, and the FFC index for the urban residents after small improvements in 2014 and 2015, decreased in 2016.

In general, the Iranian health reform had considerable impacts on the rate of the impoverished after out-of-pocket payments and seemed with limited impacts on the rates of households facing catastrophic health expenditure, and on FFC indexes. In the reform, the urban and rural residents were considered equally in improved financing of health services.

## Ethical considerations

Ethical issues (Including plagiarism, informed consent, misconduct, data fabrication and/or falsification, double publication and/or submission, redundancy, etc.) have been completely observed by the authors.

## Footnotes

1 The software confirmed by Medicine Faculty Graduate Committee, Shahed University, and was registered in the Center of Information Technology Development and Digital Media, named as Joshani-HFC.

2 The equivalent household size, *eqsize_h_*, is determined by each household size to the power 0.56, which is the estimated value from WHO studies on household survey data in 59 countries.
